# Radiosensitisation of Hepatocellular Carcinoma Cells by Vandetanib

**DOI:** 10.3390/cancers12071878

**Published:** 2020-07-13

**Authors:** Sami Znati, Rebecca Carter, Marcos Vasquez, Adam Westhorpe, Hassan Shahbakhti, Jessica Prince, Petra Vlckova, Chiara De Vellis, Zainab Bascal, Marilena Loizidou, Ricky A. Sharma

**Affiliations:** 1 University College London Cancer Institute, University College London, London WC1E 6BT, UK; rebecca.carter@ucl.ac.uk (R.C.); marcos.vasquez@ucl.ac.uk (M.V.); a.westhorpe@ucl.ac.uk (A.W.); h.shahbakti@ucl.ac.uk (H.S.); petra.vlckova.16@ucl.ac.uk (P.V.); chiara.devellis@stud.unifi.it (C.D.V.); 2 Department of Oncology, University of Oxford, Oxford OX3 7DQ, UK; jessicacprince@aol.com; 3 Scuola di Scienze Matematiche, Fisiche e Naturali, Università degli Studi di Firenze, 50121 Florence, Italy; 4 Biocompatibles UK Ltd. (A BTG International Group Company), Lakeview, Riverside Way, Watchmoor Park, Camberley, Surrey GU15 3YH, UK; zainab.bascal@btgsp.com; 5 Division of Surgery and Interventional Science, Royal Free Campus, University College London, London NW3 2QG, UK; m.loizidou@ucl.ac.uk; 6 NIHR University College London Hospitals Biomedical Research Centre, UCL Cancer Institute, University College London, London WC1E 6DD, UK

**Keywords:** vandetanib, radiotherapy, 3D models, immunocompetent, syngeneic, animal model, vascular endothelial growth factor receptor-2, angiogenesis, radiosensitiser, hepatocellular carcinoma

## Abstract

Hepatocellular Carcinoma (HCC) is increasing in incidence worldwide and requires new approaches to therapy. The combination of anti-angiogenic drug therapy and radiotherapy is one promising new approach. The anti-angiogenic drug vandetanib is a tyrosine kinase inhibitor of vascular endothelial growth factor receptor-2 (VEGFR-2) and RET proto-oncogene with radio-enhancement potential. To explore the benefit of combined vandetanib and radiotherapy treatment for HCC, we studied outcomes following combined treatment in pre-clinical models. Methods: Vandetanib and radiation treatment were combined in HCC cell lines grown *in vitro* and *in vivo*. In addition to 2D migration and clonogenic assays, the combination was studied in 3D spheroids and a syngeneic mouse model of HCC. Results: Vandetanib IC${}_{50}$s were measured in 20 cell lines and the drug was found to significantly enhance radiation cell kill and to inhibit both cell migration and invasion *in vitro*. *In vivo*, combination therapy significantly reduced cancer growth and improved overall survival, an effect that persisted for the duration of vandetanib treatment. Conclusion: In 2D and 3D studies *in vitro* and in a syngeneic model *in vivo*, the combination of vandetanib plus radiotherapy was more efficacious than either treatment alone. This new combination therapy for HCC merits evaluation in clinical trials.

## 1. Introduction

The risk of liver cancer has been steadily increasing since the end of the 20th century [1], now accounting for more than 8% of cancer-related deaths and 5% of cancer occurrence worldwide [2]. Hepatocellular carcinoma (HCC) is by far the most common liver cancer, accounting for 70–85% of total liver cancer occurrence [3], whereas Intrahepatic and Extrahepatic Cholangiocarcinoma (ICC and ECC respectively) occur less frequently. Patients with HCC are treated with curative resection if possible, or other therapies depending on staging [4,5]. Historically, targeting radiation to the liver with acceptable therapeutic ratio has been a challenge; however, as radiation is implicated in 40% of all curative cancer treatments [6], there are ongoing efforts to treat HCC with Stereotactic Body Radiation Therapy (SBRT). One approach to improving SBRT outcomes for HCC patients is the use of radiosensitisers [7], drugs which combine with radiotherapy to enhance the anti-cancer effect.

Radiosensitisers can act via intrinsic methods by directly impairing cell function and ability to survive radiation-induced damage, as is the case with platinum-based DNA chelators [8,9,10,11] and PARP inhibitors [12], both of which impair cellular DNA repair and replication. Other radiosensitisers act extrinsically by placing a toxic burden on the cell prior to radiation, as is the case with PI-103 and mTOR blockade which disrupt autophagy, leading to cell death [13]. Vandetanib is a substrate-specific tyrosine kinase inhibitor (TKI), with ten-fold selectivity for vascular endothelial growth factor receptor-2 (VEGFR-2) over off-target TKIs and secondary inhibition of the epidermal growth factor receptor (EGFR), the vascular endothelial growth factor receptor-3 (VEGFR-3) and the RE-arranged during Transfection (RET) receptor [14,15].

HCC development is often characterised by expansion of the vascular system, resulting in aberrant tumour vasculature and an immunosuppressive environment [16,17,18]. To combat this avenue of pathogenesis, treatment of HCC with the angiogenic-inhibitor class of drugs has been investigated [18,19,20] with some success. Vandetanib is unique in this class of drugs, primarily because of a high selectivity for VEGFR-2. As VEGFR-2 is the primary driver of pro-angiogenic signalling in the cell [21], selective inhibition of the receptor produces “cleaner” signal transduction [22] and leads towards vascular normalisation. Vascular normalisation is known to have a range of benefits in cancer treatment; by normalising chaotic vasculature and eliminating the hypoxic, nutrient-deprived environment fostering tumour growth, the treatment can also alter the immunosuppressive environment of the tumour by improving immune infiltration [23,24]. Vandetanib has also been shown to positively influence tumour oxygenation [25], providing one explanation for observed anti-tumour activity. Though the exact mechanism remains uncertain, previous work has repeatedly demonstrated synergy between vandetanib and radiation therapy in non-small cell lung cancer, colorectal cancer and hepatoma models [25,26,27,28,29,30]; discoveries warranting further investigation.

To evaluate vandetanib as a radiosensitiser in HCC, the effect of vandetanib-radiation combination therapy on HCC and cancer-related processes such as migration, invasion, growth and viability was studied *in vitro* with 2D and 3D models and in immunocompetent mouse models.

## 2. Results

### 2.1. VEGFR-2 Expression for Human and Mouse Epithelial and Endothelial Cell Lines

**Figure 1:**
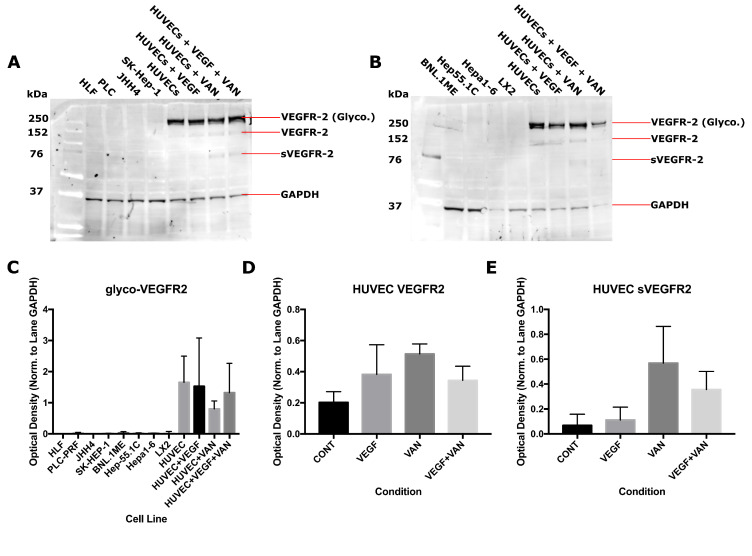
Expression status of vascular endothelial growth factor receptor-2 (VEGFR-2). VEGFR-2 expression was compared to Human Umbilical Vein Endothelial Cell (HUVEC) controls in (**A**) human and (**B**) murine cell lines. Cell extracts were labelled with VEGFR-2 antibody and Glyceraldehyde 3-phosphate dehydrogenase (GAPDH) antibody as a loading control. HUVEC cell lines were serum starved and treated with vandetanib for 24 h, then dosed with 50 ng/mL vascular endothelial growth factor (VEGF) for 20 min prior to fixation. Expression of glycosylated (**C**), native (**D**), and soluble vascular endothelial growth factor receptor-2 (VEGFR-2) (**E**) as measured by optical density is depicted.

In the normal tissue micro-environment, expression of VEGFR-2 is thought to be limited mainly to the endothelium and absent in the surrounding epithelial tissue. It is not clear how this expression changes following cellular differentiation into cancer and the possibility exists for cancer cells with VEGFR-2 expression. The expression of VEGFR-2 in human and murine cell lines was measured with western blotting to assess functional VEGFR-2 signalling and susceptibility to vandetanib and similar TKIs, both of which have implications for cancer growth and vandetanib treatment (Figure 1). VEGFR-2 status reflects the functionality of the VEGF-VEGFR-2 circuit in each cell line. The results obtained demonstrate convincing evidence of the absence of VEGFR-2 from a range of cell lines: from human epithelial cell lines PLC/PRF, HLF, and JHH4; from murine epithelial cell lines BNL.1ME, Hep55.1C, and Hepa1-6; and from endothelial associated cell lines SK-HEP-1, a cell line with a known endothelial origin [31], and LX-2, a hepatic stellate cell line and endothelial-associated pericyte [32]. VEGFR-2 expression was present in primary Human Umbilical Vein Endothelial Cells (HUVECs), indicating that they express this protein with functional post-translational modifications (PTMs), including glycosylation and the secreted soluble form following extracellular cleavage (sVEGFR2). Vandetanib treatment affected cellular expression of VEGFR-2 by modulating PTMs at the cell surface. Vandetanib-treated samples produced higher levels of the non-functional [33] native protein isoform without glycosylation as well as increased levels of the soluble receptor domain (Figure 1C,D). This pattern of expression occurred both in the presence and absence of VEGF. Importantly, release of the soluble sVEGFR-2 isoform is an indicator of angiogenic suppression [34]. Based on the blotting results, the mechanism of vandetanib activity *in vitro* can be narrowed down to intrinsic TKI-mediated disruption of VEGFR-2 activation of the RAS/RAF/MEK/ERK pathway [35] localised only to the VEGFR-2 expressing endothelium or a more functionally agnostic result owing to vandetanib effect on the cellular environment, specifically induction of autophagy and generation of ROS [36] inside the cells or the binding of the inhibitor to non-specific off-target TKs and the ensuing downstream effects.

### 2.2. IC${}_{50}$ for Vandetanib Across 20 HCC Cell Lines

**Figure 2:**
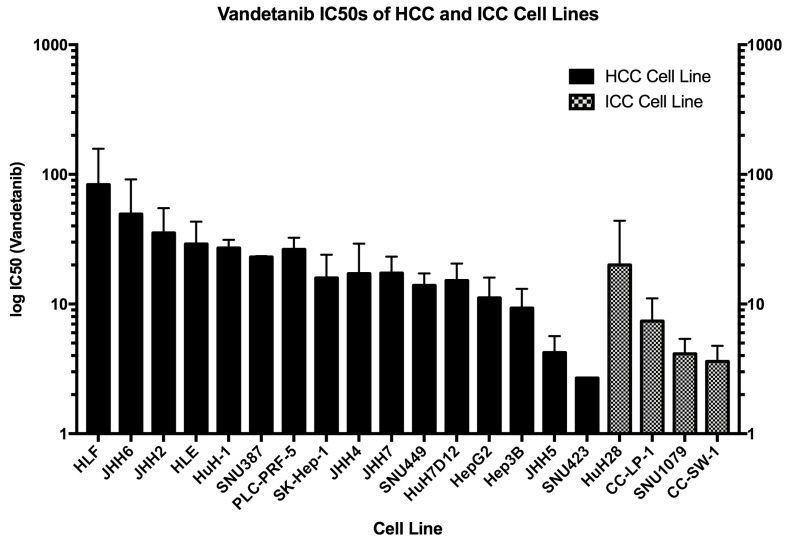
Vandetanib IC${}_{50}$ in HCC and ICC cell lines. The IC${}_{50}$ response observed in both Hepatocellular Carcinoma (HCC) and Intrahepatic Cholangiocarcinoma (ICC) cell lines demonstrated more than twenty-fold variation (range 2.7–83 μM, IQR 5.71–24.1 (19.9), error 0.25–52.5). Cell lines were tested under conditions of short exposure to drug (24 h) and measured at five days. HCC cell lines of more invasive character (HL- and JHH- cell line families) showed the least response across all cell lines.

To establish an effective dose range for vandetanib treatment *in vitro*, a series of experiments was performed on a range of liver cancer cell lines. Cells were treated with serial dilutions of vandetanib and survival was quantified to determine the Inhibitory Concentration at 50%-maximal (IC${}_{50}$). IC${}_{50}$ for short exposure (24 h) to vandetanib was measured for each cell line after five days (Figure 2) and a range of susceptibility was observed, suggesting varying levels of resistance. Cell lines known to be mutated in oncogenic RET (CC-SW-1 and HepG2) were among the more susceptible cell lines to vandetanib treatment. The IC${}_{50}$ measured did not appear to associate with sub-type in terms of donor demography or cellular derivation. Overall, ICC cell lines were more susceptible to vandetanib treatment than HCC cell lines.

### 2.3. Long Exposure to Vandetanib Sensitises HCC Cells to Radiation

**Figure 3:**
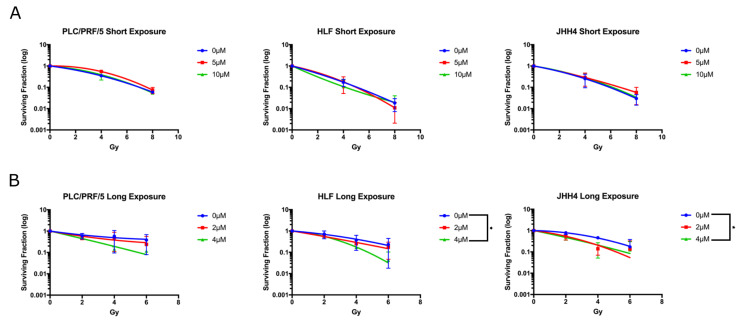
Clonogenic assays measuring sensitizing effect of vandetanib in conjunction with ionising radiation. Following treatment, response to vandetanib was observed consistently across all cell lines. (**A**) When cells were administered vandetanib at high dose (5–10 μM) for short duration (24 h), no radiosensitisation was measured. (**B**) Cells receiving a lower dose (2–4 μM) for longer exposure (72 h) showed enhancement of radiation-induced cell death. Cells treated with 5 or 10 μM vandetanib were eradicated after long exposure and were not included in the study. * RER exceeds 95% CI, ANOVA *p* ≤ 0.05.

The clonogenic radiosensitivity assay was performed to characterise the *id ipsum* ability of vandetanib to sensitise cells to radiation (Figure 3) and experiments were carried out according to two experimental protocols. In the first protocol, cells were treated with a high dose (5–10 μM) of vandetanib for a short exposure time (24 h) *in vitro*, and colony formation was used as a metric for cellular survival. Simultaneously, a second experiment of vandetanib radiosensitisation was conducted in which cells were treated with a lower dose (2–4 μM) of vandetanib for a longer exposure time (72 h) and again, cellular survival was quantified with respect to colony formation. The lower dose of 2–4 μM was selected for the second study after the initial dose of 5 and 10 μM was found to be entirely prohibitive of colony formation. When cells were administered vandetanib according to the first regime, no radiosensitisation was found. On the other hand, cells receiving the second regime of treatment did show enhancement of radiation-induced cell death, suggesting exposure longer than 24 h is necessary to observe the full radiosensitising effect. Radiation enhancement ratios (RERs) for the long exposure experiment were determined to be in the range of 1.45–1.8 for JHH4 (4 Gy + drug corresponding to 5.8–7.2 Gy dose, 95% CI) and 1.38–1.62 for HLF (4 Gy + drug corresponding to 5.4–6.4 Gy dose, 95% CI). Additionally, in the long exposure study, the HLF and JHH4 vandetanib groups both showed significance (ANOVA, *p*≤ 0.05) over controls for both drug treated groups.

### 2.4. Vandetanib and Radiation Combine to Inhibit Migration in 2D HCC Cell Culture

**Figure 4:**
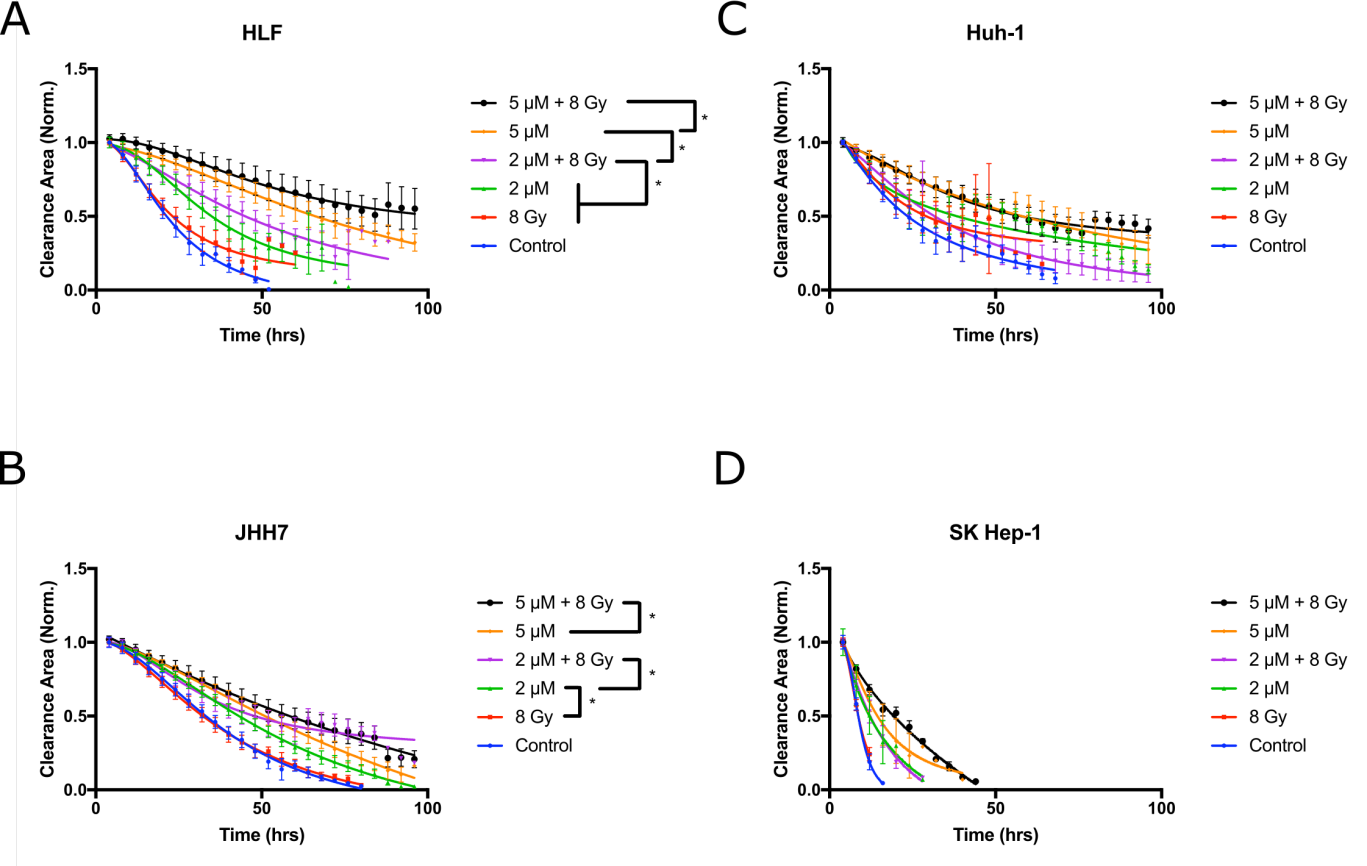
Treatment with vandetanib and radiation inhibits the migration of cells into a scratched area clearance. HCC cell lines (**A**) HLF, (**B**) JHH7, (**C**) HuH-1, and (**D**) SK-Hep-1 were plated to confluence onto 96 well plates, and a 2 mm section was cleared to generate a void. The wells were imaged every 4 h to monitor cell migration into the void. Lower values indicate faster migration and closure of the clearance. A significant, dose-dependent reduction in migration was demonstrated following vandetanib treatment (Multiple comparisons * *p* ≤ 0.05), an additive reduction in migration was observed in conjunction with radiation (non-parametric ANOVA *p* ≤ 0.05 for HLF and JHH7).

Vandetanib treatment is known to reduce migration of HCC cell lines *in vitro* [37] and we postulated that this effect would be enhanced with radiation. To test this hypothesis, the inhibition of cellular migration into a clearance was characterised with a scratch assay (Figure 4). Both vandetanib and radiation therapies were found to produce a significant, dose-dependent reduction in migration after treatment (*p* ≤ 0.05) and the combined treatment was found to produce an additive reduction in migration (*p*≤ 0.05), suggesting vandetanib inhibition of migration proceeds by alternate mechanisms to those of radiation-induced inhibition.

### 2.5. Combined Vandetanib-Radiation Invasion Inhibition of HCC Cells into Matrigel

**Figure 5:**
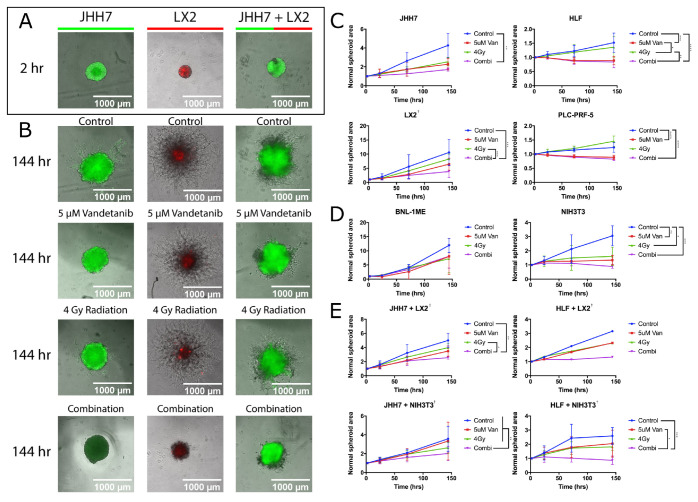
Vandetanib-radiation synergise in 3D spheroid model. HCC cell lines grown in mono-culture and co-culture 3D spheroids. (**A**,**B**) JHH7 cells fluorescently labelled green, LX2 cells fluorescently labelled red. (**A**) Reference photo of a typical time-point 0 spheroid for comparison. (**B**) Final time-point for each treatment including combination and control. (**C**) Charts depicting growth for mono-culture human spheroids. (**D**) Charts depicting growth for mono-culture murine spheroids (**E**). Charts depicting growth for co-culture spheroids. Eight models demonstrated significance, and LX2 and all co-culture models demonstrated synergy; 2-Way ANOVA * *p* ≤ 0.05, ** *p* ≤ 0.01, *** *p* ≤ 0.001, **** *p* ≤ 0.001. † CDI > 1. Point images of all time-points, cell lines and conditions included in parts (**A**,**B**) in Supplemental Figure S3. Additional point images for other cell lines also included in Supplemental Figures S5 and S6.

The Matrigel invasion assay (Figure 5) measures inhibition of cancer growth and invasion into surrounding tissue with the regular photographic measurement of a 3D cell-spheroid. When the human HCC cell line HLF was grown in 3D spheroids, significant growth reduction was observed in spheroids treated with both 5 μM vandetanib and 4 Gy radiation compared to spheroids treated with 4 Gy radiation alone (*p*≤ 0.05) or 5 μM vandetanib alone (*p* ≤ 0.001). Similarly, when the human hepatoma cell line PLC-PRF-5 was grown in 3-D, significant growth reduction was measured in spheroids treated with combined 5 μM vandetanib and 4 Gy radiation compared to control (*p* ≤ 0.001) and 5 μM vandetanib compared to control (*p* ≤ 0.001). To study the relationship between the tumour micro-environment and radiosensitivity, spheroid studies were performed with cancer cell lines grown in co-culture with fibroblast or hepatic stellate cell types. In 3D spheroid co-culture of HLF and NIH3T3 fibroblasts receiving combination treatment, invasion was significantly reduced over radiation (*p* ≤ 0.05) and vandetanib (*p* ≤ 0.0001) and likewise in 3D spheroid co-culture of JHH7 cancer cells and LX2 hepatic stellate cells, spheroid growth was inhibited in combination treatment compared to 5 μM vandetanib (*p*≤ 0.05) and 4 Gy radiation (*p* ≤ 0.005). JHH7 spheroids co-cultured with NIH3T3 and treated with combined vandetanib and radiation showed significant growth reduction compared to either single treatment (*p* ≤ 0.01 vs. 5 μM vandetanib and 4 Gy radiation) and compared to control (*p* ≤ 0.01). Similar results (Figure S6) were observed for mouse cell lines Hep55.1-C (*p*≤ 0.05 vs. control) and Hepa1-6 (*p*≤ 0.05 vs. 4 Gy radiation and control) when grown in spheroids. The murine cell line BNL1ME A.7R.1 was tested to eliminate the possibility of oversensitivity to either treatment as BNL.1ME was also cultured for tumour engraftment in the animal model (see Section 2.6). Of the remaining models studied, LX2 and each co-culture model demonstrated synergy by coefficent of drug interaction (CDI [38], see Appendix A.1). For additional results including all Figure 5 time points see supplemental (Figures S3, S5 and S6).

### 2.6. Vandetanib-Radiation Combination Inhibits Tumour Growth in an Immunocompetent Mouse Model

**Figure 6:**
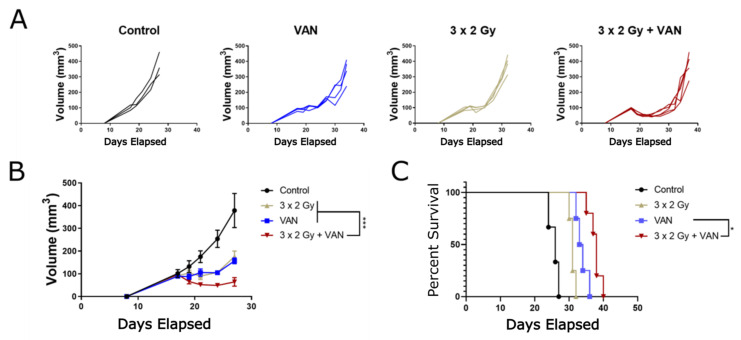
Immunocompetent mouse model of HCC shows significant anti-tumour effect of vandetanib-radiation treatment over single therapy. Immunocompetent BALB/C mice were engrafted with BNL.1ME tumours and administered fractionated radiotherapy on days 17, 18, and 19 and/or vandetanib treatment on days 17–24. Tumour growth was measured in three dimensions until culling. (**A**) Tumour volume for all mice by treatment cohort. (**B**) Mean tumour volume. (**C**) Survival curve. Combined treatment was significant (*p* ≤ 0.001) with respect to tumour volume and significant (*p* ≤ 0.05) with respect to overall survival against both regimes and control. Volume growth curves are expressed as mean ± SEM and analyzed by extra sum-of-squares F test. Survival is represented by a Kaplan–Meier plot. Log-rank test. * *p* ≤ 0.05, *** *p* ≤ 0.005.

Past studies have characterised the effect of vandetanib in animal models and shown radiation enhancement [25,26,27,28,29,30]. To better characterise the anti-tumour effect of radiation fractions comparable to those used conventionally in the clinic to treat cancer patients, an immunocompetent mouse study was carried out with fractionated 2 Gy doses of radiotherapy. In this study, the effect of combination therapy on a tumour grown in an immunocompetent microenvironment was examined (Figure 6) with a syngeneic model and the BNL.1ME A.7R.1 cell line. This cell line was previously studied in a 3D model (Figure 5D). In the single-treatment groups, tumours experienced a growth delay characteristic of tumour treatment, with significant (3 × 2 Gy + VAN vs. 3 × 2 Gy *p* ≤ 0.005, 3 × 2 Gy vs. VAN *p* ≤ 0.0001) size reduction compared to control. When treated with either vandetanib or radiation alone, the tumours remained stationary for seven days, at which point the growths recurred. In combination therapy, the tumours initially shrunk in size compared to the starting measurements and growth remained inhibited longer than the single treatment groups. Tumour growth reduction was significant versus control (*p* ≤ 0.0001), and regression in size was seen until day 10, concurrent with the end of vandetanib treatment, when the tumours began to exhibit signs of growth recurrence.

## 3. Discussion

Vandetanib is a multi- tyrosine kinase inhibitor specifically targeted to angiogenic and growth promoting receptors on the exterior surface of the plasma membrane. *In vitro* radiosensitivity assays suggest a prolonged exposure is required for an intrinsic radiosensitisation effect, as evidenced by the results from the clonogenic assay (Figure 3) which demonstrated a clear functional difference between cells exposed to a high dose of vandetanib for a short 24 h period and those exposed to a low dose for a longer 72 h duration. Cells treated with low dose vandetanib for three days demonstrated significant radiosensitisation and radiation enhancement ratios of 1.4 or beyond, whereas those in the second, shorter exposure group did not show any enhancement. These results are in line with those published elsewhere [29] and with the outcomes of vandetanib treatment in 3D *in vitro* models which also proceeded over a longer time scale and produced similar radiosensitivity effects after combination vandetanib-radiation treatment. One explanation for these findings is anticipated by literature results indicating vandetanib induces autophagy [36] and supported by experimental observations of a similar autophagic response to both vandetanib and radiation (Figure S4). These results suggest cells postpone cell death for longer than 24 h, allowing them to recover once the drug is removed after the short 24 h time frame. Cells exposed to vandetanib for an extended 72 h time frame may die before the drug is removed, meaning the cells begin dying from autophagic exhaustion at some point between the conclusions of the short exposure and long exposure time frames, relative to drug dose. This may suggest local treatment with vandetanib, which has the advantage of prolonged high local dose avoiding systemic toxicity [39,40,41], may have a greater benefit than systemic treatment by increasing radiosensitivity and autophagy.

Vandetanib effects on migration were demonstrated to be dose-dependent and an additive effect was observed with combination vandetanib-radiation treatment. This would indicate the two proceed by alternative, non-competitive mechanisms, in contrast to the effects measured in the other *in vitro* assays which suggest some level of interaction. Vandetanib as a TKI may exert a much stronger effect over the tumour micro-environment than a direct intrinsic effect on the cancer cells themselves. As evidenced by VEGFR-2 expression *in vitro* (Figure 1) and supported by literature results demonstrating a lack of VEGFR-2 expression in HCC tissue stained for the receptor [42,43,44], the cancer cells do not express the receptors of the VEGF circuit, explaining the less pronounced effect seen with *in vitro* models. For these cases, the full effect of vandetanib-induced inhibition of signalling pathways is absent, and it must be concluded this *in vitro* vandetanib treatment would have a less pronounced effect than when the complete signalling circuit is recapitulated *in vivo*. The *in vitro* results do suggest vandetanib would affect cancer cell migration and vascular organisation, among other effects.

In the 3D spheroid assays, vandetanib was shown to disrupt the means of growth and invasion essential to cancer proliferation, and in the case of the LX2 cell line grown alone as well as for all co-cultures tested, vandetanib was found by CDI [38] to exhibit a synergistic effect in combination with radiotherapy. It comes as no surprise this effect is most visible in the co-culture studies as it seems likely the cell signalling pathways disrupted by vandetanib are important to the growth of tumour-supporting stromal cells as well as the metastasis and invasion of those cells. Furthermore, as the cell lines tested *in vitro* were found to be negative for VEGFR-2 expression, the mechanism of action must be external to the VEGFR-2 signalling pathway. As much of the effect of vandetanib rests upon altering the tumour microenvironment, primarily via vascular normalization [24,45], the drug response measured *in vitro* raises the issue of how the cells are inhibited without vandetanib binding to the target receptor. We suggest autophagy following from vandetanib treatment to be critical to the observed inhibition (Supplemental Figure S4), but research into the effect of vandetanib would benefit from *in vitro* studies with bio-mimetic models of endothelial vasculature [46] to better characterise this phenomenon.

In immunocompetent mouse studies, combination treatment was found to be additive with mean survival for the combination therapy equivalent to the sum total survival reached by both treatments alone (Figure 6C). By tumour growth alone (Figure 6B), a significant (*p* ≤ 0.001) sub-additive [47] effect was measured. This result differs from those observed in previous immunocompromised studies [26,27,28,29,30], which measured effects beyond addition. It is important to note, BNL.1ME spheroids were shown to be highly treatment resistant in 3D models, and this may partially explain the discrepancy. Furthermore, the experimental regimes in the literature administered either larger doses of radiation or more numerous 2 Gy fractions, or both, and were conducted with immunocompromised mouse models. From our immunocompetent study we found simple growth delay and tumour volume in the combined treatment to be significantly improved over both single treatments and the control treatment and found the reduction in tumour size to begin immediately after starting vandetanib treatment (day 9). Critically, we found the presence of vandetanib in the tumour environment severely hindered tumour growth for the entire duration of drug administration (day 9–17). When taken into consideration with the *in vitro* results, particularly the clonogenic assay which also found the duration of vandetanib exposure to be an important factor for achieving radiosensitisation, it would appear the radiosensitisation occurs at a stage after the initial binding and inhibition of the receptor. The dynamics of *in vivo* treatment with vandetanib have been shown to alter the physical properties of the tumour microenvironment, by increasing oxygenation immediately following vandetanib treatment [25] and the anti-angiogenic class of drugs in general have been shown to decrease tumour vessel permeability and interstitial fluid pressure [45,48]. Interestingly, when combined with radiotherapy this period of increased oxygenation would coincide with increased tumour perfusion leading to fluid extravasation from the tumour and a reduction in interstitial pressure and hypoxia in the tumour microenvironment [45,48,49,50,51] due to radiation. Additionally, the well documented [23,24] vascular normalisation effect of angiogenic therapy is another means of anti-tumour effect due to vandetanib therapy. This effect may be compounded by direct action of the drug on immune system modulators, as there is evidence of VEGFR2 expression among immunosuppressive populations of T cells [52]. Targeting VEGFR2 with anti-angiogenesis treatment may, alongside physical alterations to the cancer tissue, offer another mechanism by which combined treatment could be potentiated *in vivo*.

It is apparent from the *in vitro* results vandetanib significantly enhances the effect of radiation on cell viability as seen with the clonogenic assay, migration in the scratch assay, and growth and invasion in the spheroid study. The effects of combined treatment, on not only these various components of the tumour machinery but on the ability of the tumour to develop, are most visible in the animal model where a reduction in size persisted for the duration of treatment and additive effects to overall survival were found. The sum total of these results indicates vandetanib enhances HCC treatment when administered concomitantly with radiation and suggests a potentially significant benefit could be derived from the development of combined vandetanib-radiation therapy for the treatment of HCC patients.

## 4. Materials and Methods

### 4.1. Cell Lines

The human cell lines used in this study are shown below in Table 1. Other factors considered during analysis relating to donor characteristics are also shown in Table 1. Murine cell lines are listed in Table 2.

### 4.2. Monolayer Cell Culture

Cell lines were grown under standard cell culture conditions (humidified, 37 C, 21% O${}_{2}$, 5% CO${}_{2}$) in Dulbecco’s Modified Eagle Medium (DMEM) + 10% Fetal Bovine Serum (FBS) and 1% Penicillin/streptomycin (all procured from Gibco, Waltham, MA, USA). HUVEC cells were grown in EGM-Plus (Lonza, Basel, Switzerland). Cells were passaged regularly at 60–80% confluency.

### 4.3. Plasmid Transfection

Plasmids containing a pcDNA3-EGFP (Doug Golenbock: Addgene plasmid # 13031) or pQCXI Neo DsRed-LC3-GFP (David Sabatini: Addgene plasmid # 31183 [53]) retroviral vector were transformed into competent TG1 E. coli strain bacteria according to protocol [54]. Transformed bacteria were plated onto selective agar plates (MP biomedicals, Morocco) and cultured for 24 h before inoculation in LB Agar (Sigma, Dorset, UK) and purification with Qiagen Maxi-Prep or Midi-Prep kits (Qiagen, Hilden, Germany). After plasmid purification, human cell lines were transfected with FuGene HD Transfection Reagent (Bio-rad, Hercules, CA, USA) via the corresponding transfection protocol. Two 5 cm dishes (Corning, Corning, NY, USA) were seeded with 5 × 10${}^{5}$ cells and cultured overnight under cell culture conditions. After 24 h, one dish was transfected with plasmid according to protocol, with the second dish acting as a control. At 48 h following addition of plasmid, both dishes were treated with selective agent until the control cells were completely dead. Transfected cells were then removed from selective media and grown under normal cell culture conditions. Fluorescent protein expressing cells were then isolated with Fluorescence Assisted Cell Sorting to obtain the maximum yield of cells expressing the fluorescence vector. The transfected cell lines were used in the experiments depicted in Figure 5A,B. pcDNA3-EGFP was a gift from Doug Golenbock (Addgene plasmid # 13031). pQCXI Neo DsRed-LC3-GFP was a gift from David Sabatini (Addgene plasmid # 31183; http://n2t.net/addgene:31183; RRID:Addgene_31183)

### 4.4. X-ray Irradiation of In Vitro Samples

An XStrahl (Camberley, UK) Small Animal Radiotherapy Research Platform (SARRP), S/N 525722, was used for *in vitro* sample irradiation. X-ray emissions at effective energies of 65.2 keV [55] were delivered from a Comet MXR225/22 X-Ray tube source set to operate at 220 kV and 13 mA with beam filtration via an integral 0.8 mm Be window and additional 0.15 mm Cu filter. Samples were irradiated on a 1.5 cm thick Perspex movable platform and lead shielding totalling 5 mm thickness was placed directly on the plates for additional shielding as required. All samples were irradiated 30 min after drug treatment, in line with previous work [26] indicating 30-min irradiation post-treatment to be optimal.

Dosimetry for the SARRP was measured using a PTW 30012 Farmer ionisation chamber (PTW-UK Ltd., Lincolnshire, UK) and a UNIDOS E electrometer (PTW-UK Ltd., Lincolnshire, UK) corrected and calibrated to the National Physical Laboratory (Teddington, UK) primary standard. Reference conditions: Open field; source to surface distance (SSD), 33 cm; phantom thickness, 5.5 cm; detector’s geometric centre at 2 cm depth, 35 cm; and half-value layer, 0.658 mm Cu.

### 4.5. Western Blotting

HCC cells were seeded in DMEM and serum starved for 24 h prior to harvesting. HUVEC cell lines were serum starved and treated with vandetanib for 24 h, then dosed with 50 ng/mL VEGF for 20 min prior to fixation. Cell culture samples were washed with PBS and frozen at −80 C before being lysed on ice with RIPA Lysis buffer. Protein concentration was measured by Bradford assay against BSA standard, and 30 μg of protein were loaded onto the lanes of a 10-well 4–12% Bis-Tris NuPAGE gel (Invitrogen, Carlsbad, CA, USA) with NuPAGE loading buffer and run at 125 V for 90 min before transfer onto a PVDF membrane, blocking with LI-COR blocking buffer (LI-COR Biosciences, Lincoln, NE, USA), and staining with primary anti-VEGFR-2 (Cell Signalling Technology, Beverly, MA, USA, #2479) and anti-GAPDH (Cell Signalling Technology, #97166) before secondary labelling and imaging on an Odyssey CL-X imaging system (LI-COR Biosciences). Quantification measurements were performed with the ImageJ (NIH, Bethesda, MD, USA) [56] Optical Density Plugin and all sample measurements were first normalised to lane GAPDH levels.

### 4.6. IC${}_{50}$ Determination

Cell line IC${}_{50}$s for vandetanib were obtained for a panel of 20 cell lines. Cells were plated on 96-well plates at a concentration of 15,000 cells/mL (3000 cells/well) prior to treatment with vandetanib (serially diluted from 160 μM to 0.25 μM) for 24 h. IC${}_{50}$ was measured five days following drug treatment via cellular metabolism of resazurin. Cell culture media was replaced with Fluorobrite^TM^ media containing 10 μg/mL resazurin. Resazurin metabolism into fluorescent resorufin was monitored for up to 4 h, at which point fluorescence emission at 590 nm was measured with a Perkin Elmer Envision 2103 plate reader (Waltham, MA, USA), and the dose corresponding to 50% survival was determined from the survival curves using Graphpad Prism (San Diego, CA, USA).

### 4.7. Clonogenic Survival

HCC cells from tissue culture cell lines were trypsinised and counted, and 2000 cells at a concentration of 250 cells/mL were plated on 10 cm dishes (Corning) for 24 h before addition of vandetanib in 0.1% DMSO and/or treatment with irradiation. Drug exposure was maintained for either 24 or 72 h, depending on the experiment, before media was changed to remove the drug from the cellular environment. Drug controls were treated with 0.1% DMSO. Radiation controls received mock irradiation to maintain equivalent cell culture conditions. After ten days, the cells were fixed in methanol and stained with 0.4% Methylene Blue in Methanol or 330 μM Crystal Violet in 2% Ethanol (Sigma) and colonies were counted by two independent observers. Statistical analysis was performed in Graphpad Prism, where regression analysis was performed to derive radiation enhancement ratios. Significance was determined with 2-way ANOVA.

### 4.8. Migration Assay

HCC cells were plated to confluence on a 96-well plate and a 2 mm section of confluency was cleared with an Incucyte Woundmaker (Essen Biosciences, Essen, Germany) across the centre of the monolayer. Cells were treated with vandetanib in 0.1% DMSO or vehicle (0.1% DMSO) and/or irradiation. Migration into the clearance was imaged at regular 4 h intervals with a Nikon Biostation (Tokyo, Japan) and quantified with ImageJ software [56]. Significance was determined via one-way ANOVA with multiple comparisons (Tukey) using Graphpad Prism. Additive effect was determined via comparison of confidence intervals from the ANOVA non-parametric test in Graphpad Prism. Synergy was evaluated with CDI [38].

### 4.9. 3D Spheroid Cell Culture

Spheroids were formed according to a standard spheroid protocol [57]. HCC cells were plated on 96 well plates at 10,000 cells per well in 200 μL DMEM + 10% FBS, centrifuged at 250× *g* for 5 min and allowed to form aggregates under normal cell culture conditions for a period of three days. Following aggregation, the cells were placed on ice, the media was removed and replaced with 100 μL Matrigel matrix (Corning) diluted with DMEM to a protein concentration of 4–6 mg/mL. DMSO or vandetanib treatment was added at indicated concentrations to the Matrigel mixture on ice during dilution and prior to setting. Cells were irradiated immediately following drug treatment. Spheroids were then allowed to grow at normal cell culture conditions for one week with growth and invasion into the gel matrix measured at regular intervals. Spheroid growth was quantified with the TASI OrganoSeg segmentation software [58]. Significance figures were measured with two-way ANOVA in Graphpad Prism. Synergy was evaluated with CDI [38] (see Appendix A.1).

### 4.10. Immunocompetent Animal Model

All experiments were performed on immunocompetent BALB/c mice (Charles River, Wilmington, MA, USA). Animal experiments were conducted with approval from UK Home Office (licence 70/8861) in accordance with the European Commission Directive 2010/63/EU (European Convention for the Protection of Vertebrate Animals used for Experimental and Other Scientific Purposes), the UK Home Office (Scientific Procedures) Act (1986), and the National Institutes of Health Guide for the Care and Use of Laboratory Animals, with project approval from UCL Biological Services. Animals were housed in a temperature-controlled facility with 12 h on–12 h off light cycle and provided water and laboratory rodent food *ad libitum*.

### 4.11. BNL.1ME Tumour Engraftment and Treatment

The BNL.1ME mouse cell line was selected from among three hepatic murine cell lines (Table 2) due to rapid growth properties, low immunogenicity and consistency in forming lesions. The cell line was replated after undergoing three *in vivo* passages in BALB/C mouse and allowed to reach 60–80% confluency before harvest under sterile conditions and subcutaneous injection of 5 × 10${}^{5}$ cells in a volume of 50 μL of PBS into the right flank of isoflurane-anesthetised mouse. Tumour growth was measured every other day. Radiation and/or drug treatment began nine days after injection. Animals were administered 25 mg/kg vandetanib in DMSO or DMSO as control for ten days. Radiation group animals were irradiated on three consecutive days receiving one fraction of 2 Gy radiation each day. Survival was measured from injection point. Significance was evaluated with Log-Rank test in Graphpad Prism. Mice were monitored daily for signs of ill health and radiation toxicity including loss of >10% body weight, hunched posture, loss of active movements, or skin, gastro-intestinal or lung changes. Mice were euthanised by cervical dislocation at humane endpoints including clinical signs of ill health or radiation toxicity, persistent tumour ulceration for >24 h, maximal mean tumour diameter of 1.5 cm, and at predetermined study endpoints. There was no unanticipated mortality due to drug or radiation treatment.

## 5. Conclusions

In 2D and 3D studies of radiosensitisation in multiple HCC cell lines, combined treatment with vandetanib and radiation was more beneficial than either treatment alone, reducing cell viability, migration, and invasion *in vitro*. The effect of the combination therapy was most marked *in vivo*, where a syngeneic mouse model of HCC found combination-treated tumours to reduce in size significantly more than those receiving single treatment, a reduction that persisted for the duration of vandetanib treatment and led to an additive benefit to overall survival when compared to either treatment alone. Collectively, these findings help to characterise vandetanib as a radiosensitiser for HCC and suggest the need for clinical trial exploration of this treatment combination.

## Figures and Tables

**Table 1:** Human cell lines used for research.

Cell Line	Source/Access. No.	Donor Patient	Derivation	Cell Type	Etiology
SK-Hep-1	ATCC HTB-52	52 M	Adenocarcinoma	endothelial/ mesenchymal [31]	
Hep G2	ECACC 85011430	15 M Caucasian	HCC	epithelial	
Hep3B	ECACC 86062703	8 M Black	Hepatocyte carcinoma	epithelial	
JHH2	JCRB1028	57 M Japanese	Hepatoma		
JHH4	JCRB0435	51 M Japanese	HCC		
JHH5	JCRB1029	50 M Japanese	HCC		
JHH6	JCRB1030	57 F Japanese	HCC	epithelial	
JHH7	JCRB1031	53 M Japanese	HCC	epithelial	HepB
HLE	JCRB0404	68 M	Hepatoma		
HLF	JCRB0405	68 M	Hepatoma	epithelial	
PLC-PRF-5	ATCC CRL-8024		Hepatoma	epithelial	HepB
Huh1	JCRB0199	53 M Japanese	HCC	epithelial	HepB
Huh7-D12	ECACC 010427	12 M	HCC	epithelial	
SNU423	ATCC CRL-2238	40 M Asian	pleo. HCC	epithelial	HepB
SNU387	ATCC CRL-2237	41 F Asian	pleo. HCC	epithelial	HepB
SNU449	ATCC CRL-2234	52 M Asian	HCC	epithelial	HepB
SNU1079	B. Dwyer ‡		ICC		
Huh28	B. Dwyer ‡		ICC		
CC-LP-1	B. Dwyer ‡		ICC		
CC-SW-1	B. Dwyer ‡		ICC		
LX-2	K. Rombouts *§*			Hepatic Stellate Cell [32]	
HUVEC	Lonza CC-2935		Umbilical Vein	endothelial	

‡ Cell line obtained courtesy of B. Dwyer at the University of Edinburgh, Edinburgh, United Kingdom. *§* Cell line obtained courtesy of Prof. K. Rombouts at UCL, London, United Kingdom.

**Table 2:** Murine Cell lines used for research.

Cell Line	Source/Accession No.	Mouse Background	Deriv.
Hep-55.1C	CLS-GmbH	C57BL/6 Mouse	HCC
Hepa1-6	CLS-GmbH	C57BL/6J Mouse	HCC
BNL1ME A.7R.1	ATCC TIB-75	BALB/C Mouse	HCC/ epithelial
NIH3T3	ATCC CRL-1658	mouse embryo	fibroblast

## Supplementary Materials

The following are available online at https://www.mdpi.com/2072-6694/12/7/1878/s1, Figure S1: Example IC50 curves, Figure S2: Clonogenic Drug Titration, Figure S3: All timepoints from Figure 5, Figure S4: Autophagy Assay, Figure S5: Additional Human Spheroid Results, Figure S6: Additional Murine Spheroid Results, Videos S1–S4: Scratch Assay Migration under different conditions, Videos S5–S12: 3D spheroid assay of murine cell lines.

## Abbreviations

The following abbreviations are used in this manuscript:

HCC	Hepatocellular Carcinoma	ICC	Intrahepatic Cholangiocarcinoma
ECC	Extrahepatic Cholangiocarcinoma	SBRT	Stereotactic Body Radiotherapy
TKI	Tyrosine Kinase Inhibitor	PTM	Post-translational Modification
VAN	Vandetanib	PARP	Poly ADP Ribose Polymerase
DNA	Deoxyribonucleic Acid	VEGF(R)	Vascular Endothelial Growth Factor (Receptor)
EGF(R)	Epidermal Growth Factor (Recptor)	RET	REarranged during Transfection Receptor
mTOR	Mammalian Target of Rapamycin	GAPDH	Glyceraldehyde 3-phosphate dehydrogenase
2D	Two Dimensional	3D	Three Dimensional
RER	Radiation Enhancement Ratio	IC${}_{50}$	Inhibitory Concentration at 50% Maxima
p	probability value	CI	Confidence Interval
IQR	Interquartile Range	CDI	Coefficient of Drug Interaction
ANOVA	ANalysis of VAriance	MDPI	Multidisciplinary Digital Publishing Institute
SI units were used unless otherwise stated.	

## Appendix A. Mathematical Formulae

### Appendix A.1. Coefficient of Drug Interaction

Interaction [47] was measured with the Coefficient of Drug Interaction (CDI) [38] defined as:

(A1) $$CDI=\frac{\frac{\mu_{Comb}}{\mu_{Cont}}}{\frac{\mu_{rad}}{\mu_{Cont}}\times \frac{\mu_{van}}{\mu_{Cont}}}$$

### Appendix A.2. Volume Calculation for In Vivo Experiment

(A2) $$V=\frac{l\times w\times h\times \pi }{6}$$
