# Supplementary figures and images for: Radiosensitisation of Hepatocellular Carcinoma Cells by Vandetanib
Source: Cancers (Basel). 2020 Jul 13;12(7):1878. doi: 10.3390/cancers12071878 (PMC7408860; doi:10.3390/cancers12071878)

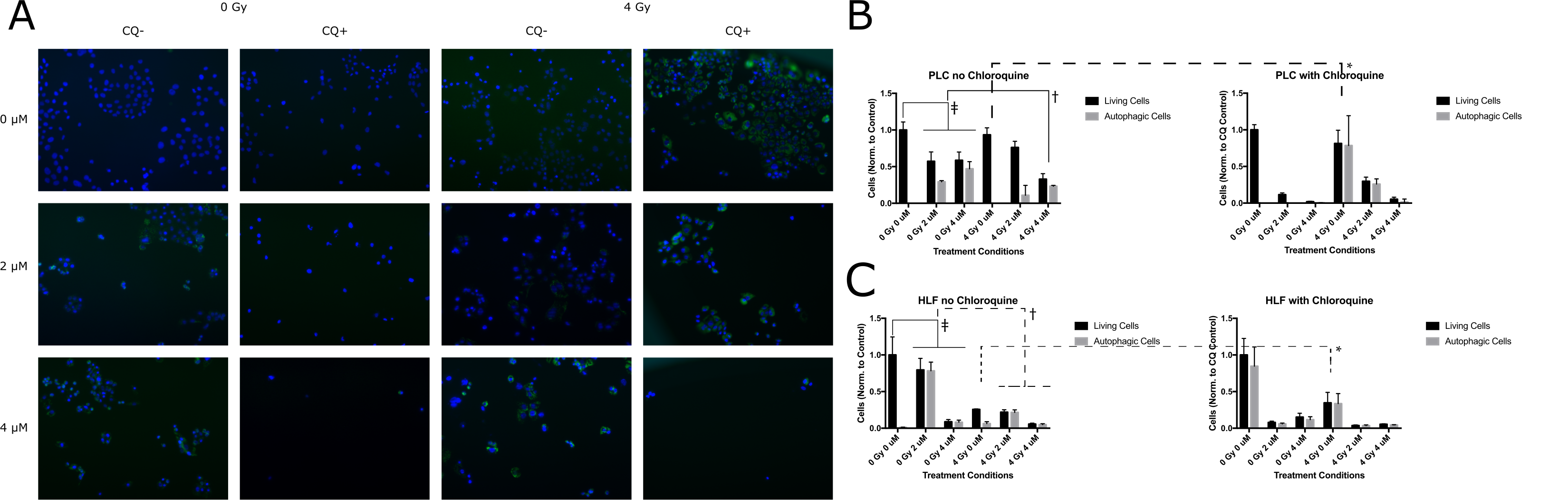

Supplement: Supplementary file 1 [file cancers-12-01878-s001.zip › Supplemental/autophagy--.png]

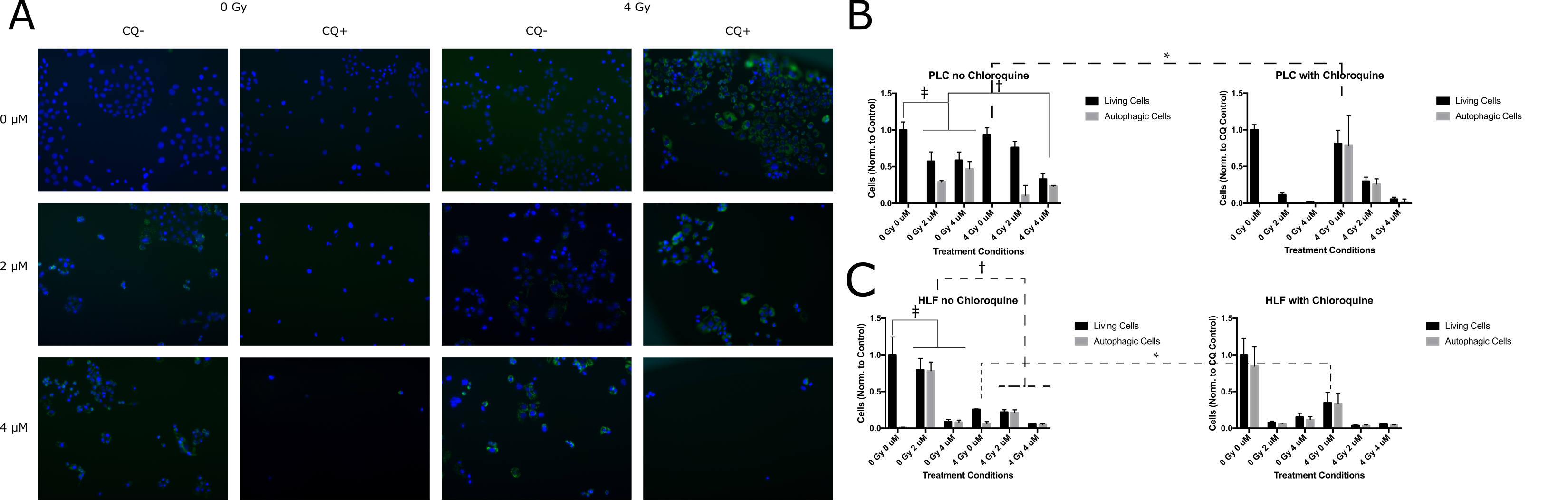

Supplement: Supplementary file 1 [file cancers-12-01878-s001.zip › Supplemental/autophagy.png]

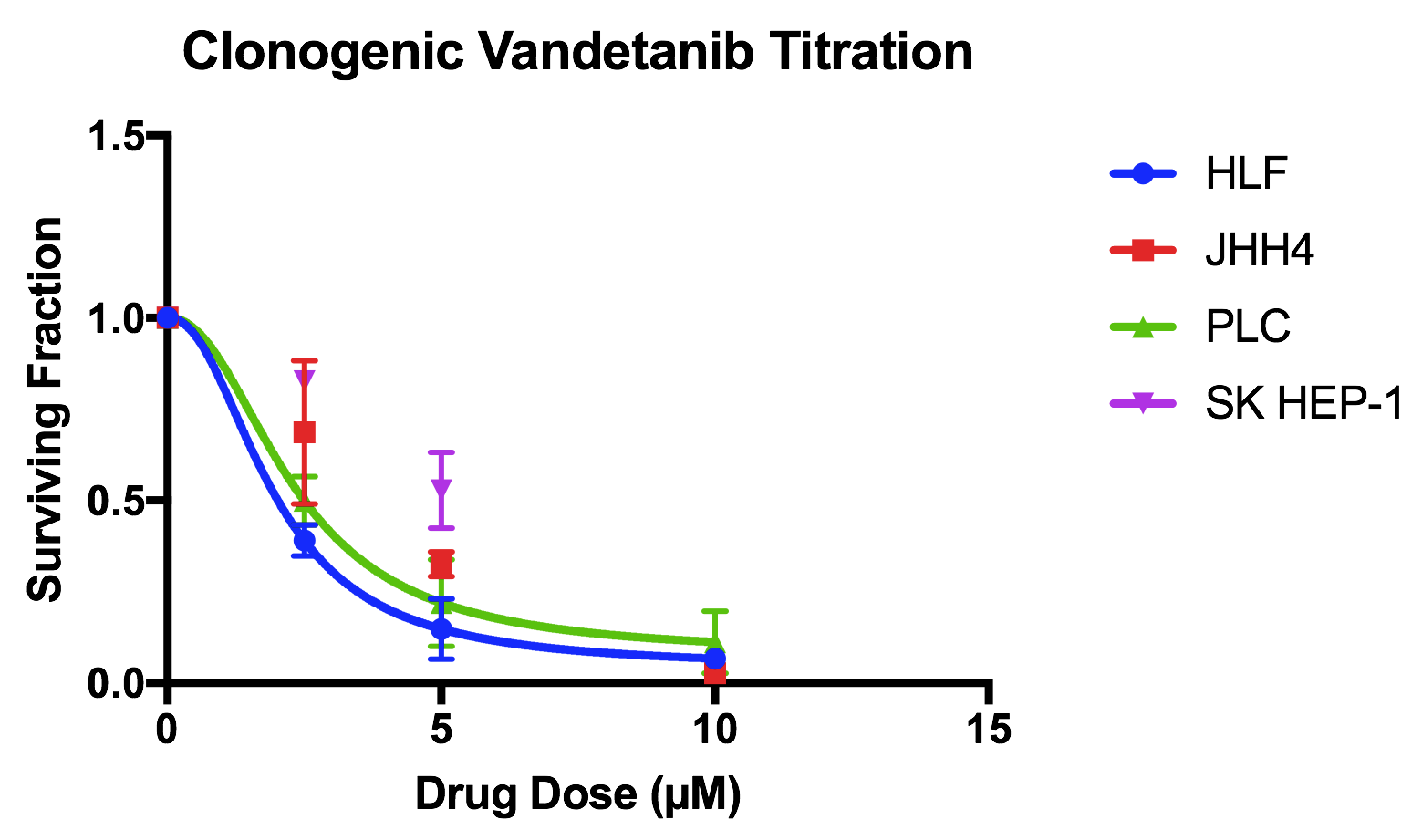

Supplement: Supplementary file 1 [file cancers-12-01878-s001.zip › Supplemental/Clono_supp.png]

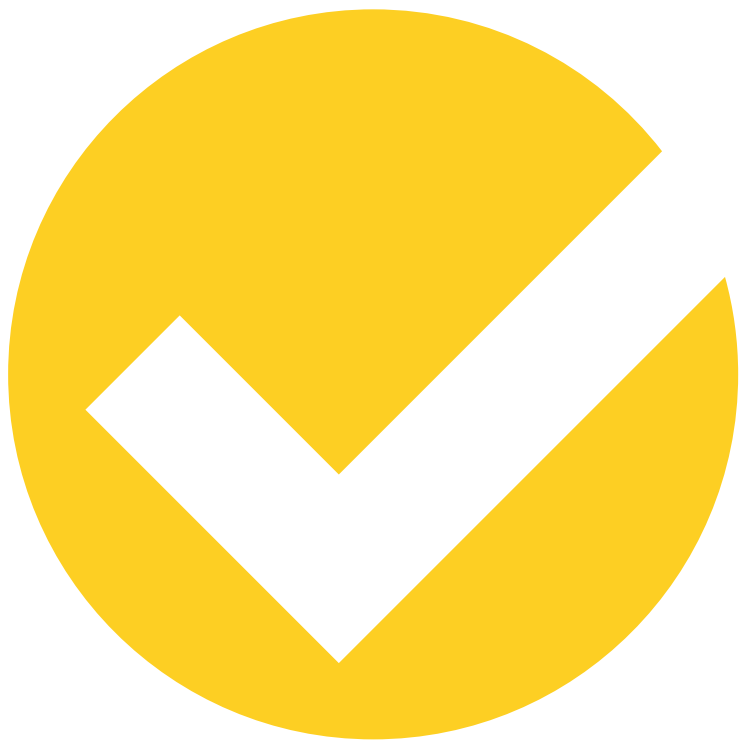

check for  
updates

Supplement: Supplementary file 1 [file cancers-12-01878-s001.zip › Supplemental/Definitions/logo-updates.pdf]

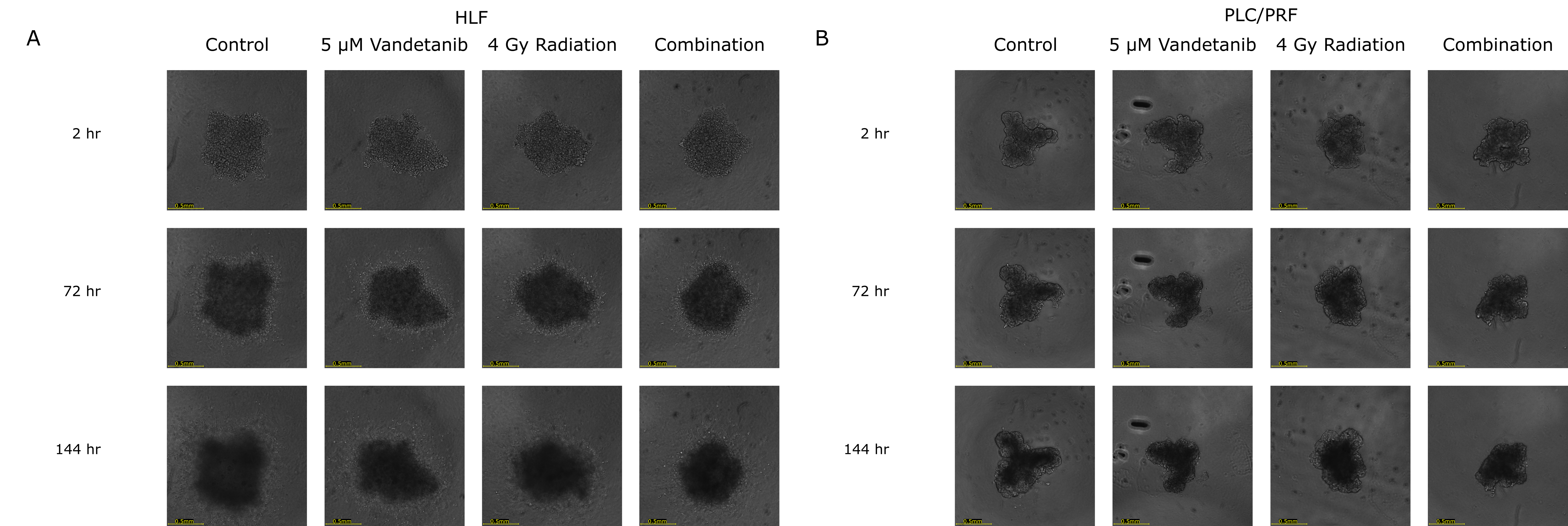

Supplement: Supplementary file 1 [file cancers-12-01878-s001.zip › Supplemental/human.png]

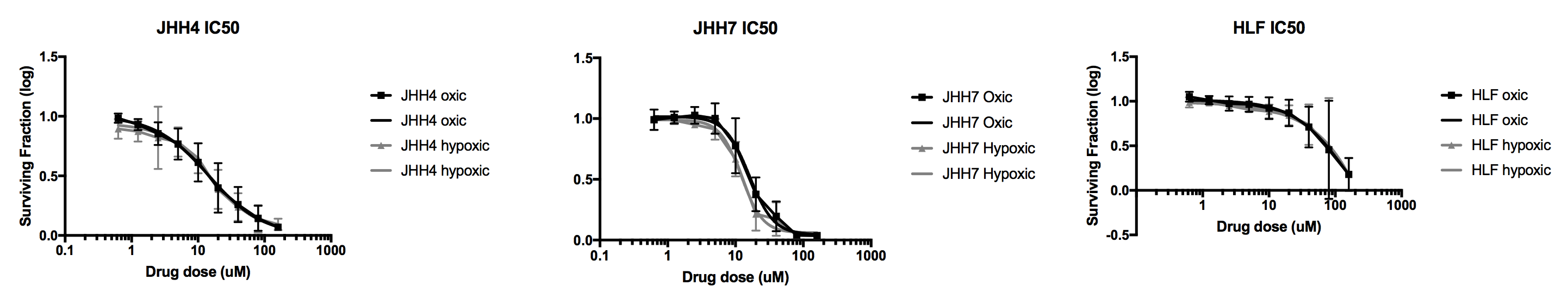

Supplement: Supplementary file 1 [file cancers-12-01878-s001.zip › Supplemental/IC50_supp.png]

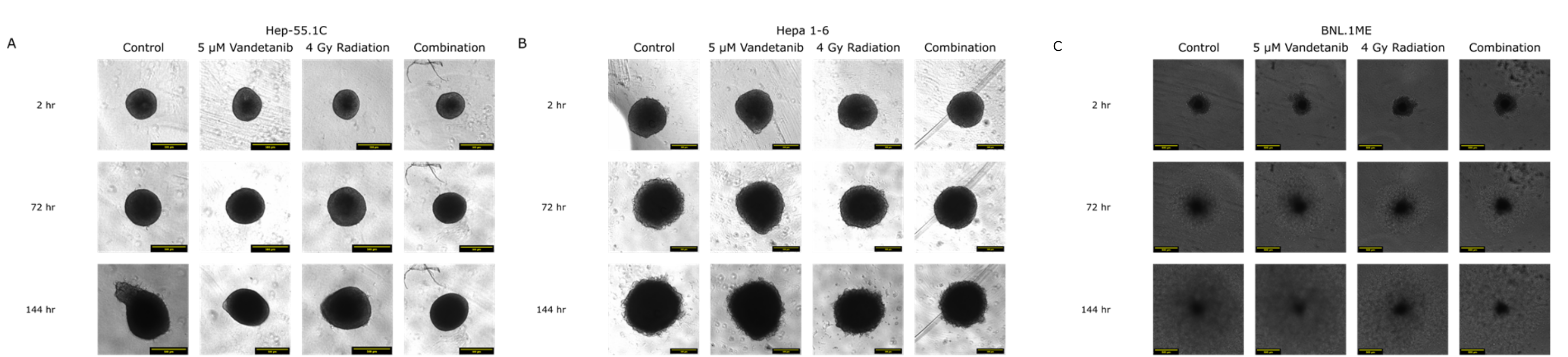

Supplement: Supplementary file 1 [file cancers-12-01878-s001.zip › Supplemental/mouse.png]

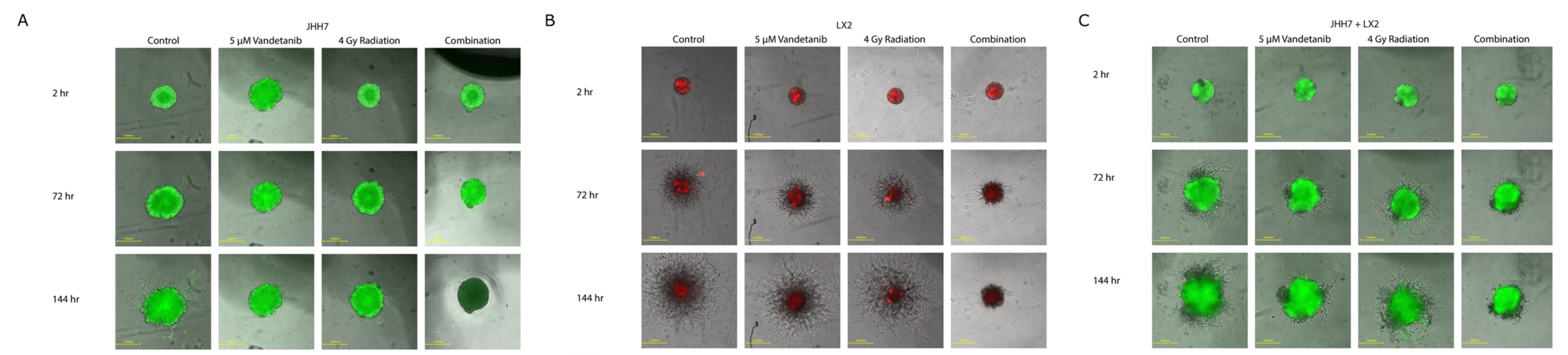

Supplement: Supplementary file 1 [file cancers-12-01878-s001.zip › Supplemental/timepoints.png]
